# Positive/Negative Allosteric Modulation Switching in an Umami Taste Receptor (T1R1/T1R3) by a Natural Flavor Compound, Methional

**DOI:** 10.1038/s41598-018-30315-x

**Published:** 2018-08-07

**Authors:** Yasuka Toda, Tomoya Nakagita, Takatsugu Hirokawa, Yuki Yamashita, Ayako Nakajima, Masataka Narukawa, Yoshiro Ishimaru, Riichiro Uchida, Takumi Misaka

**Affiliations:** 1 Department of Applied Biological Chemistry, Graduate School of Agricultural and Life Sciences, The University of Tokyo, 1-1-1 Yayoi, Bunkyo-ku, Tokyo 113-8657 Japan; 2 Department of Agricultural Chemistry, Faculty of Agriculture, Meiji University, 1-1-1 Higashimita, Tama-ku, Kawasaki, Kanagawa 214-8571 Japan; 3 Department of Cell Biology, Graduate School of Medicine, Kyoto University, Yoshida-Konoe-cho, Sakyo-ku, Kyoto 606-8501 Japan; 4 Molecular Profiling Research Center for Drug Discovery (molprof), National Institute of Advanced Industrial Science and Technology (AIST), Tokyo Waterfront Bio-IT Research Building 2-4-7 Aomi, Koto-ku, Tokyo 135-0064 Japan; 5 Research and Development Division, Kikkoman Corporation, 399 Noda, Noda, Chiba 278-0037 Japan

## Abstract

Taste is a vital sensation for vertebrates, enabling the detection of nutritionally important substances or potential toxins. A heteromeric complex of two class C GPCRs, T1R1 and T1R3, was identified as the umami (savory) taste receptor. Amino acids and 5′-ribonucleotides are well known to be natural ligands for human T1R1/T1R3. In this study, we reveal that methional, which is a familiar flavor component in foods, is an allosteric modulator of T1R1/T1R3. Receptor expression experiments showed that methional served as a positive allosteric modulator (PAM) of human T1R1/T1R3 and functioned as a negative allosteric modulator (NAM) of mouse T1R1/T1R3. Although amino acids and 5′-ribonucleotides bound to the extracellular domain of T1R1, the use of interspecies chimeric receptors demonstrated that methional interacted with the transmembrane domain of T1R1. Site-directed mutagenesis and molecular modeling showed that methional could potentially bind at two distinct sites in the transmembrane domain of T1R1 and that the amino acid residues in the bottom of the allosteric pocket engendered the switch between the PAM and NAM modes, which could contribute to switching the binding position of methional. These results may be applicable for elucidating the molecular mechanisms underlying ligand recognition by other class C GPCRs.

## Introduction

As umami not only makes food palatable but also helps to reduce the NaCl levels in foods, the demand for novel modulators of umami taste has increased^1^. Through dozens of studies, various substances, such as peptides^2^, nucleotide derivatives^3^, and Maillard-reaction products^4^, were identified as umami molecules^1^. In vertebrates, umami taste is sensed by a heteromeric complex of two class C G-protein-coupled receptors (GPCRs), T1R1 and T1R3^5^. Recently, there have been tremendous advances in the discovery of novel modulators for GPCRs that do not bind to the orthosteric ligand binding site but instead bind to an alternatively located binding site (allosteric site)^6^. Thus, we expected that there should be various allosteric modulators of human T1R1/T1R3 (hT1R1/hT1R3) among savory compounds that were discovered by sensory tests. However, only 5′-ribonucleotides, such as inosine 5′-monophosphate (IMP) and guanosine 5′-monophosphate (GMP), as well as an artificial substance, “S807”, have been shown to interact with hT1R1/hT1R3 in an allosteric manner as umami molecules^7^. One of the reasons for this paucity of ligands is the difficulty of establishing a sensitive and accurate assay system for hT1R1/hT1R3.

Methional is a familiar flavor component in foods such as tomatoes^8,9^, cheese^10,11^, and soy sauce^12^. Although its meaty aroma has been reported to evoke an umami (savory) taste^13,14^, the effect of methional on the peripheral taste system has not been elucidated. Here, we evaluated the activity of methional against hT1R1/hT1R3 using a cell-based high-throughput assay system that we previously established using calcium-sensitive photoprotein reporters^15,16^.

## Results

### Methional and its structural analogs act as allosteric/ago-allosteric modulators of human T1R1/T1R3

**Figure 1:**
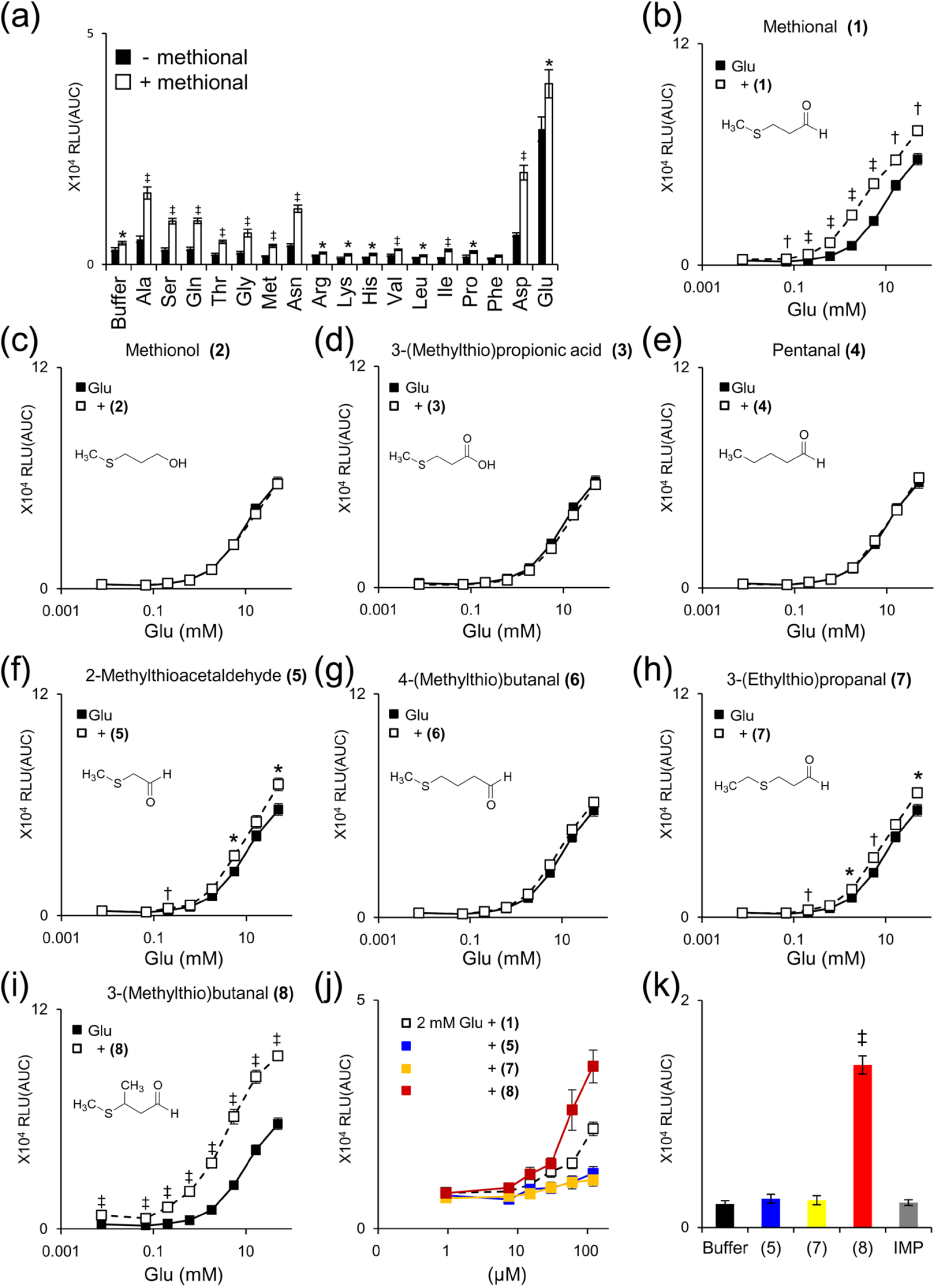
Methional and its structural analogs enhanced the responses of human T1R1/T1R3 to amino acids. (**a**) Methional enhanced the responses of hT1R1/hT1R3 to various amino acids. HEK293T cells coexpressing hT1R1/hT1R3 together with hG16gi3 were separately stimulated with 10 mM l-Glu or 50 mM concentrations of each amino acid except for l-Glu in the absence or presence of 120 μM methional. Significant differences between amino acid responses with and without methional were analyzed using Student’s *t* test (*p < 0.05; ^†^*p* < 0.01; ^‡^*p* < 0.001). (**b–i**) Activities of methional and its structural analogs with respect to hT1R1/hT1R3. Dose-dependent responses to l-Glu were obtained in the presence and absence of 120 μM of methional and each of its analogs. Significant differences between l-Glu responses with and without methional or its analogs were analyzed using Student’s *t* test (*p < 0.05; ^†^*p* < 0.01; ^‡^*p* < 0.001). (**j**) Dose-dependent responses to methional and its analogs were evaluated in the presence of 2 mM l-Glu. (**k**) 3-(Methylthio)butanal **(8)** behaved not only as a modulator but also as an agonist for hT1R1/hT1R3. HEK293T cells coexpressing hT1R1/hT1R3 together with hG16gi3 were stimulated with 120 μM of each methional analog or IMP. Values represent the mean ± SE of the RLU (AUC) of 6 recorded wells. Significant differences from the response to buffer were analyzed using a one-way ANOVA followed by Dunnett’s test (^‡^*p* < 0.001).

Humans have a strong umami taste response to monosodium glutamate and weak umami taste response to several other amino acids, such as monosodium aspartate and l-alanine (l-Ala)^17^. In accordance with these sensations, hT1R1/hT1R3 exhibits the strongest response to l-glutamate (l-Glu) among the proteinogenic amino acids^5^. Methional significantly enhanced responses to all 17 amino acids tested, except l-phenylalanine (*p* = 0.08), in hT1R1/hT1R3-expressing cells (Fig. 1a).

To identify the key chemical and structural features of methional for activating hT1R1/hT1R3, we examined seven structural analogs of methional (Fig. 1b–i). All of the analogs and methional were examined up to a concentration of 120 μM because the cellular responses were suppressed in a non-specific manner by methional and some analogs at higher concentrations. Among the seven analogs tested, neither methionol **(2)**, 3-(methylthio)propionic acid **(3)**, nor pentanal **(4)** enhanced l-Glu responses, suggesting that an aldehyde and alkylthio group are critical functional groups for activating hT1R1/hT1R3. 2-Methylthioacetaldehyde **(5)**, 3-(ethylthio)propanal **(7)**, and 3-(methylthio)butanal **(8)** significantly enhanced l-Glu responses, while 4-(methylthio)butanal **(6)** exhibited no significant effect. Among these active compounds, 3-(methylthio)butanal **(8)** and methional exhibited the strongest activities (Fig. 1j), suggesting that the optimum structure to activate hT1R1/hT1R3 is an aldehyde with a methylthio group at C-3.

**Figure 2:**
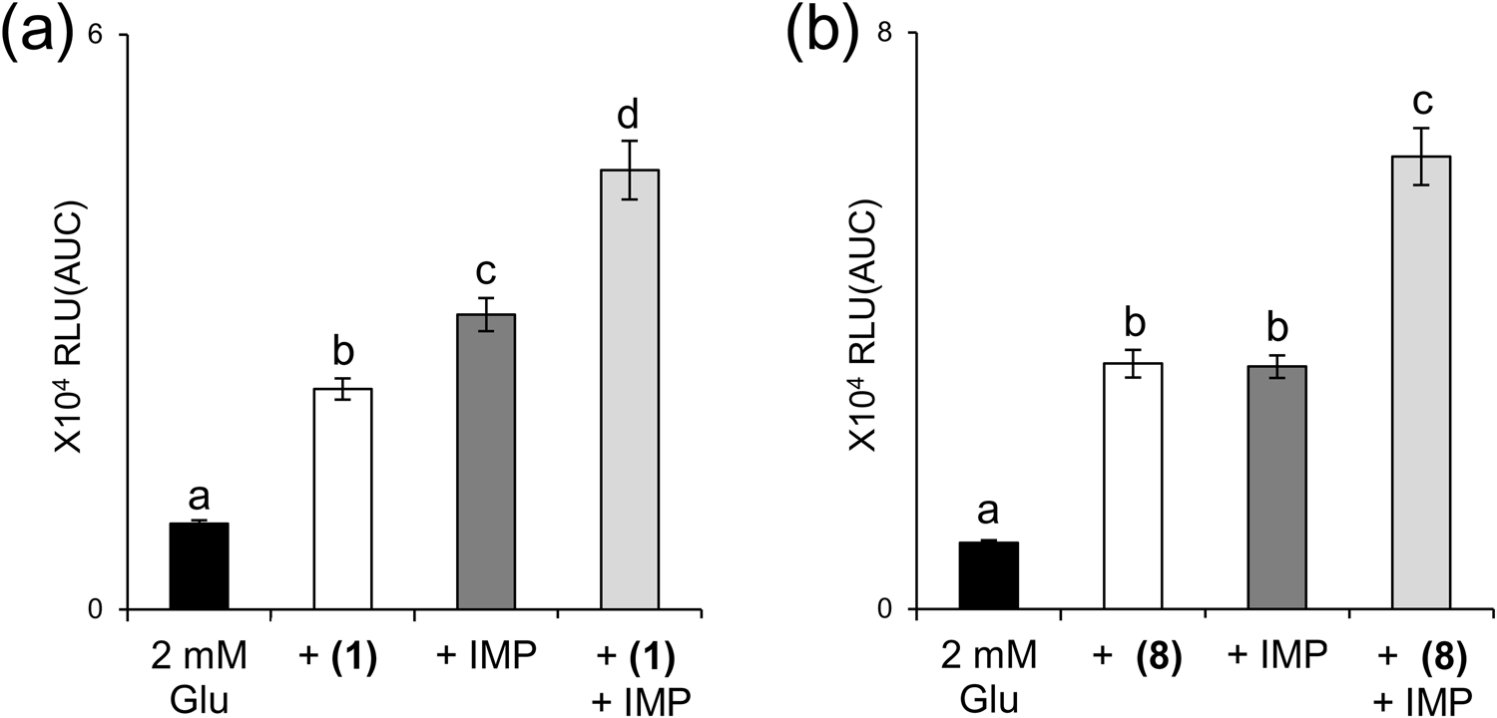
Methional and 3-(methylthio)butanal **(8)** enhanced l-Glu responses in the presence of IMP. (**a**) HEK293T cells coexpressing hT1R1/hT1R3 together with hG16gi3 were stimulated with 2 mM l-Glu in the absence or presence of 120 μM methional **(1)**, 120 μM IMP, or both. (**b**) HEK293T cells coexpressing hT1R1/hT1R3 together with hG16gi3 were stimulated with 2 mM l-Glu in the absence or presence of 120 μM 3-(methylthio)butanal **(8)**, 120 μM IMP, or both. Values represent the mean ± SE of the RLU(AUC) of 6 recorded wells. Means followed by a different letter are significantly different, as analyzed by Tukey’s test (p < 0.05).

Methional and 3-(methylthio)butanal **(8)** elicited weak but significant responses when applied alone to hT1R1/hT1R3-expressing cells (Fig. 1a,k). These results indicated that methional and 3-(methylthio)butanal **(8)** function as both PAMs and weak agonists for hT1R1/hT1R3. Methional and 3-(methylthio)butanal **(8)** enhanced l-Glu responses even when applied together with IMP (Fig. 2).

### Identification of the determinant residues for methional activity in T1R1/T1R3

**Figure 3:**
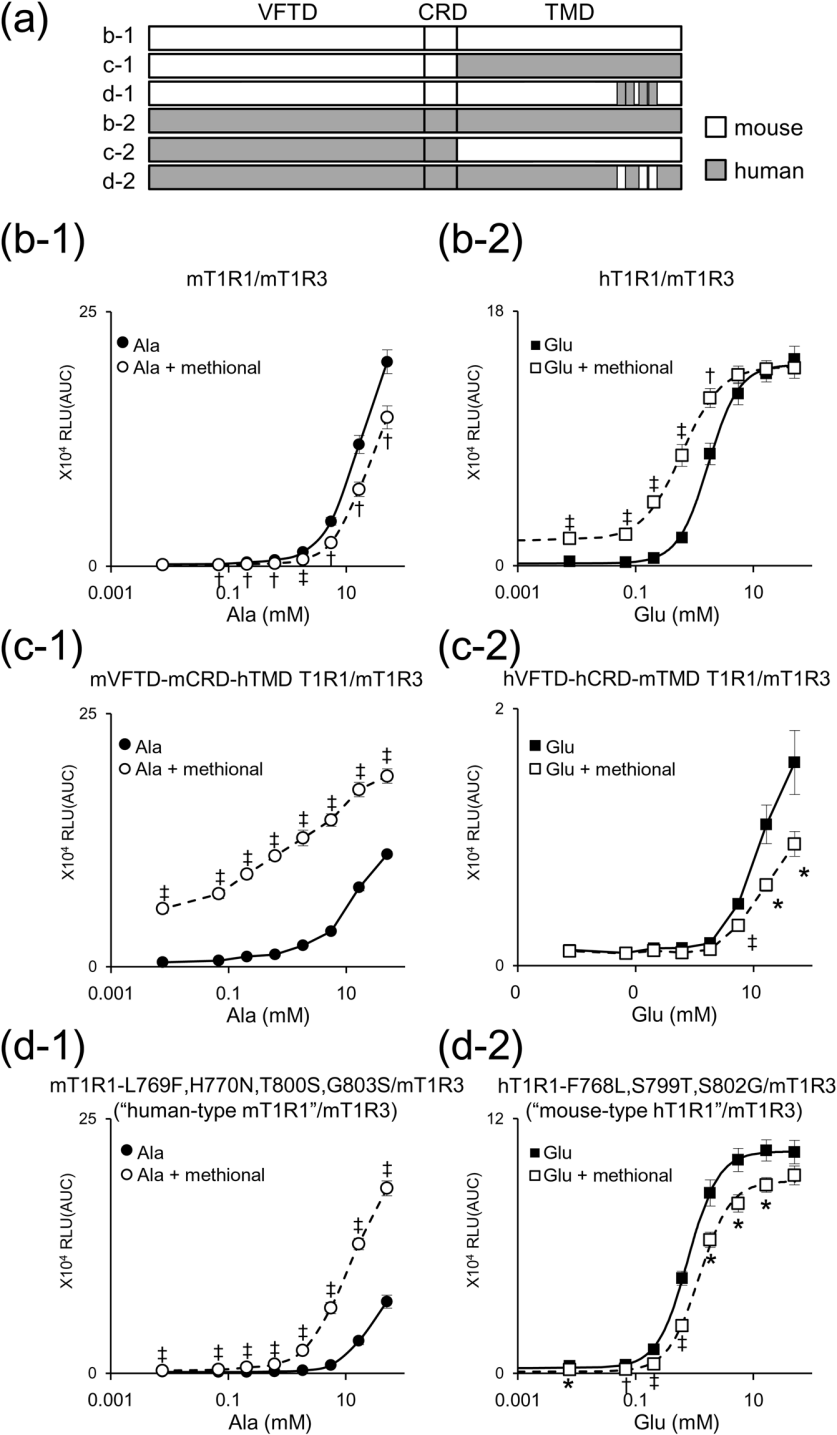
Identification of residues that engendered a switch in the PAM/NAM mode of methional activity. (**a**) T1R1 chimeras were designed to contain human (gray) and mouse amino acid sequences (white). (**b–d**) Dose-dependent responses to l-Ala (**b-1**,**c-1**,**d-1**) or l-Glu (**b-2**,**c-2**,**d-2**) were obtained for each T1R1 paired with mouse T1R3. Significant differences between amino acid responses with and without 120 μM methional were analyzed using Student’s *t* test (*p < 0.05; ^†^*p* < 0.01; ^‡^*p* < 0.001). Values represent the mean ± SE of the RLU(AUC) of 6 recorded wells. The EC_50_ and *E*_max_ values of hT1R1/mT1R3 (**b-2**) and of mouse-type hT1R1/mT1R3 (**d-2**) are shown in Supplementary Tables S1 and S2, respectively.

**Figure 4:**
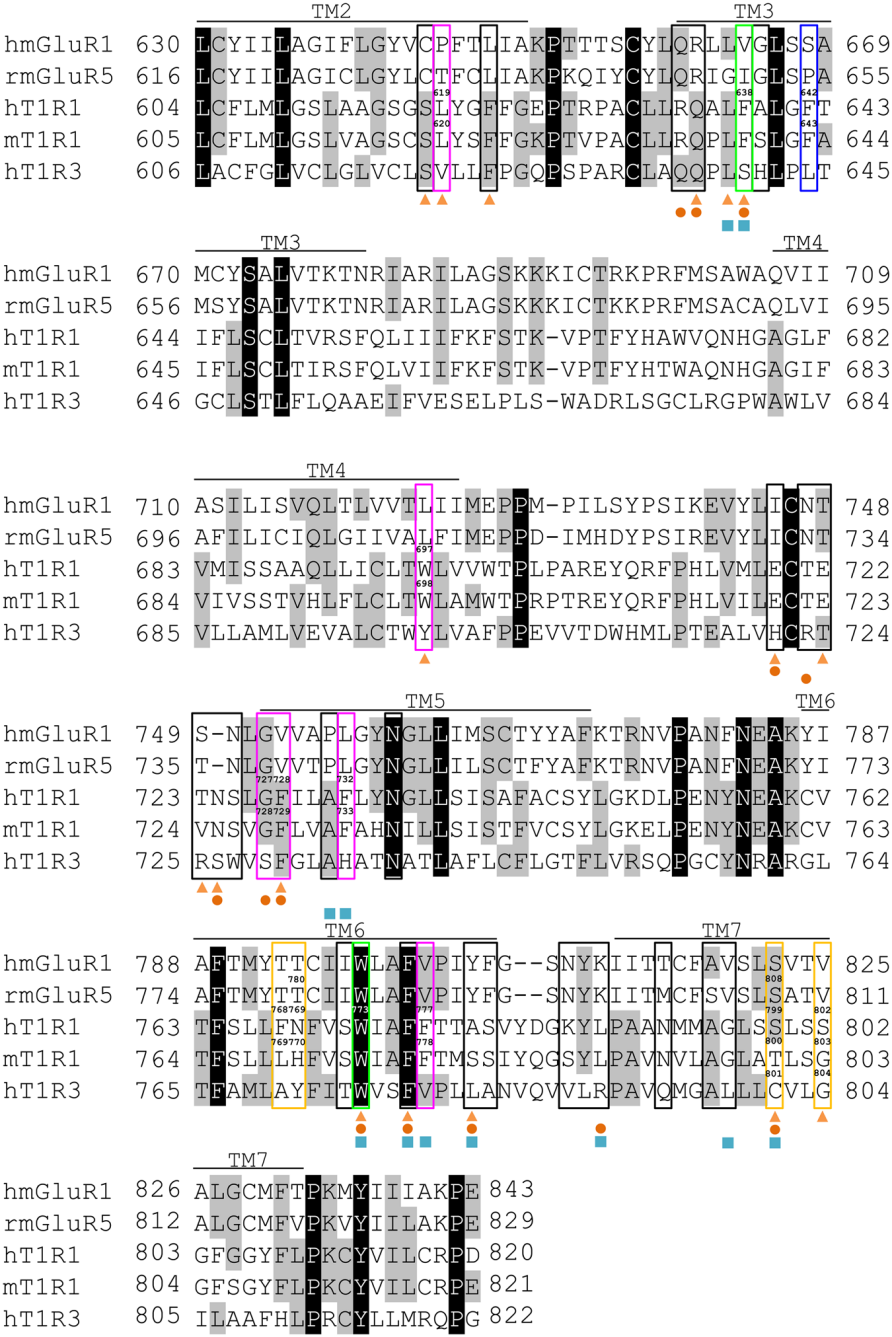
Sequence alignment of the TMD of human mGluR1, rat mGluR5, human T1R1, mouse T1R1, and human T1R3. Amino acid sequences around TM2 to TM7 are shown. The residues that were mutated in this study are framed by colored lines. The residues conferring PAM activity are outlined in magenta, those conferring NAM activity are outlined in blue, those responsible for PAM/NAM mode switching are outlined in yellow, and those responsible for the microswitch of receptor activation are outlined in green. The residues that did not show significant effects are in black. The residues that have been reported to be crucial for the activities of sweeteners and/or an inhibitor of human T1R3 are marked below the amino acid sequences with triangles (NHDC), circles (cyclamate), and squares (lactisole)^21^.

T1Rs often exhibit different ligand selectivity among vertebrate species^5,18,19^. Therefore, we next examined methional activity using mouse T1R1/T1R3 (mT1R1/mT1R3). As mT1R1/mT1R3 was only slightly activated by l-Glu, we instead used l-Ala, which is a potent agonist of the mouse receptor^5,15^. Intriguingly, methional significantly reduced l-Ala responses in mT1R1/mT1R3 (Fig. 3b-1). To dissect the molecular determinants that contributed to this unique interspecies difference, we examined the methional activity of the mixed receptor pair of human and mouse T1R1/T1R3. Methional acted as a PAM for human T1R1 paired with mouse T1R3 (Fig. 3b-2), showing that the T1R1 subunit is a crucial determinant of methional activity. T1Rs consist of three domains: a large extracellular VFTD, small extracellular cysteine-rich domain (CRD), and seven-transmembrane domain (TMD)^20^. To reveal which domain is crucial for switching PAM/NAM activities, we investigated human-mouse chimeric receptors by exchanging their TMDs. Methional served as a PAM for the heteromeric receptor in which the TMD of human T1R1 was introduced into mT1R1/mT1R3 (Fig. 3c-1). Conversely, methional acted as a NAM for the receptor in which the TMD of mouse T1R1 was introduced into hT1R1/mT1R3 (Fig. 3c-2). These data strongly suggest that the TMD of T1R1 is the key domain for switching the PAM/NAM activities of methional. Further analysis using chimeric receptors and point mutants identified four residues (h/m; F768/L769, N769/H770, S799/T800, and S802/G803) that, collectively, were sufficient to switch the PAM/NAM activities among the 52 non-conserved residues between human and mouse T1R1-TMD (Fig. 4). Introducing four human amino acids at the corresponding positions of mouse T1R1 (“human-type mT1R1”; mT1R1-L769F, H770N, T800S, and G803S) completely converted methional from a NAM to a PAM (Fig. 3d-1). In the case of human T1R1, introduction of three mouse amino acids (“mouse-type human T1R1”; hT1R1-F768L, S799T, and S802G) was sufficient to switch the action of methional from a PAM to a NAM (Fig. 3d-2).

**Figure 5:**
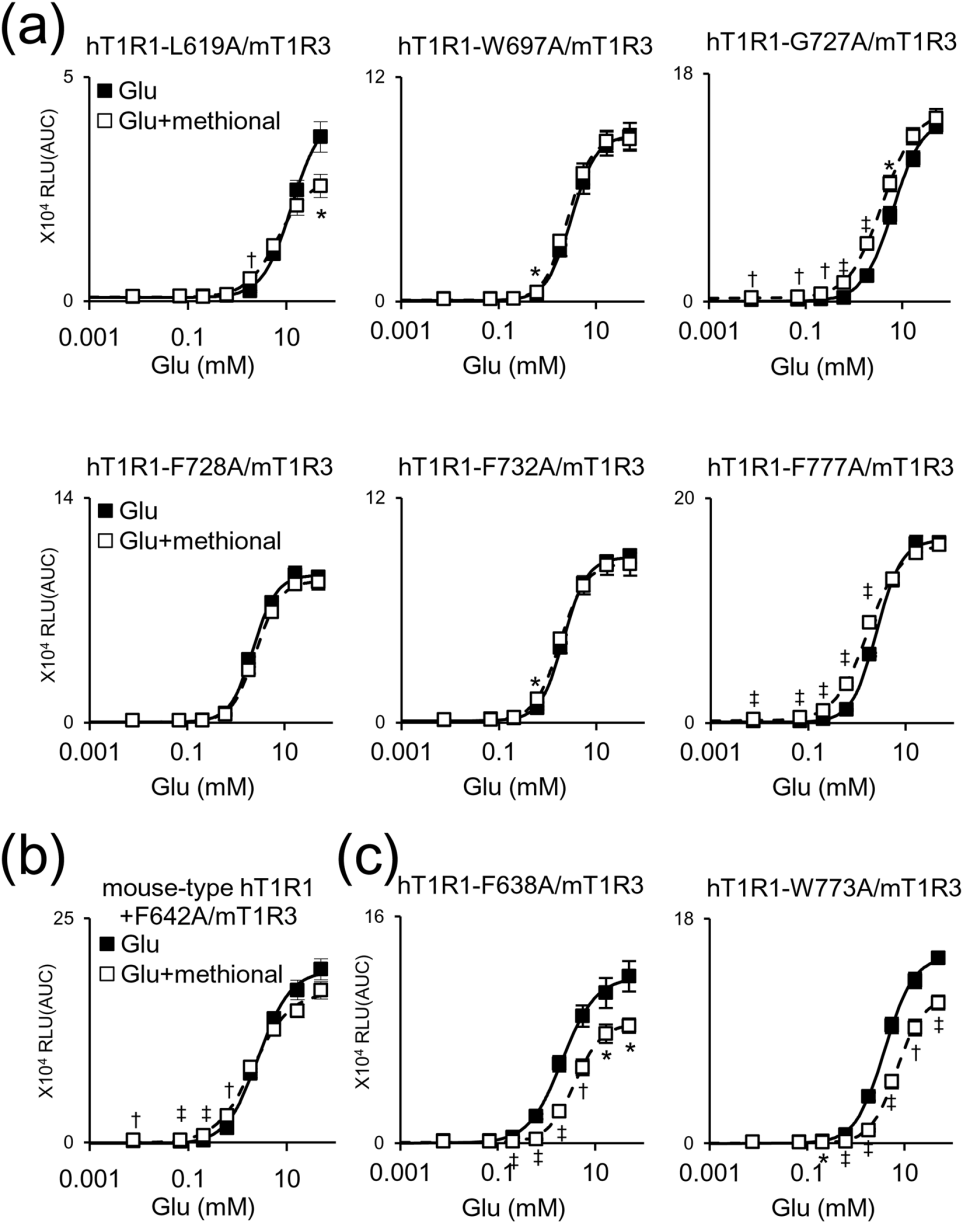
Residues that conferred the PAM and NAM activities of methional in T1R1. Dose-response curves were obtained to l-Glu for each human T1R1 mutant paired with mouse T1R3. (**a**) Of the 32 residues examined (Fig. 4), an alanine substitution in each of the six residues in hT1R1 caused either an abolition or a decrease in the PAM activity of methional. (**b**) An alanine substitution in mouse-type hT1R1 caused a decrease in the NAM activity of methional. (**c**) An alanine mutation in each of two residues in hT1R1 swapped the PAM and NAM activity of methional. Values represent the mean ± SE of the RLU (AUC) of 6 recorded wells. Significant differences between l-Glu responses with and without 120 μM methional were analyzed using Student’s *t* test (*p < 0.05; ^†^*p* < 0.01; ^‡^*p* < 0.001). The EC_50_ and *E*_max_ values of the receptors described here are shown in Supplementary Table S1 (**a**,**c**) and Table S2 (**b**), respectively.

Next, we sought to identify the residues that confer the PAM and NAM activities of methional by examining the corresponding residues that are crucial to the activities of either sweeteners (neohesperidin dihydrochalcone (NHDC) and cyclamate) and/or a sweet and umami taste inhibitor (lactisole) in human T1R3^21–23^. As most of the target residues were identical between human and mouse T1R1 (Fig. 4), we introduced an alanine mutation at each of these residues in hT1R1/mT1R3. Among the 32 residues that we examined, three point mutations (W697A, F728A, and F732A) completely abolished methional’s activity as a PAM, and another three mutations (L619A, G727A, and F777A) partially reduced this activity (Fig. 5a and Supplementary Table S1). In hT1R1-L619A, methional also caused a significant decrease in the efficacy (*E*_max_) of l-Glu, while the other five alanine substitutions did not affect the efficacy of l-Glu (Supplementary Table S1). All six mutations also resulted in decreased activity of methional as an agonist of hT1R1/mT1R3, which was observed at a low concentration of l-Glu (Figs 3b-2 and 5a).

**Figure 6:**
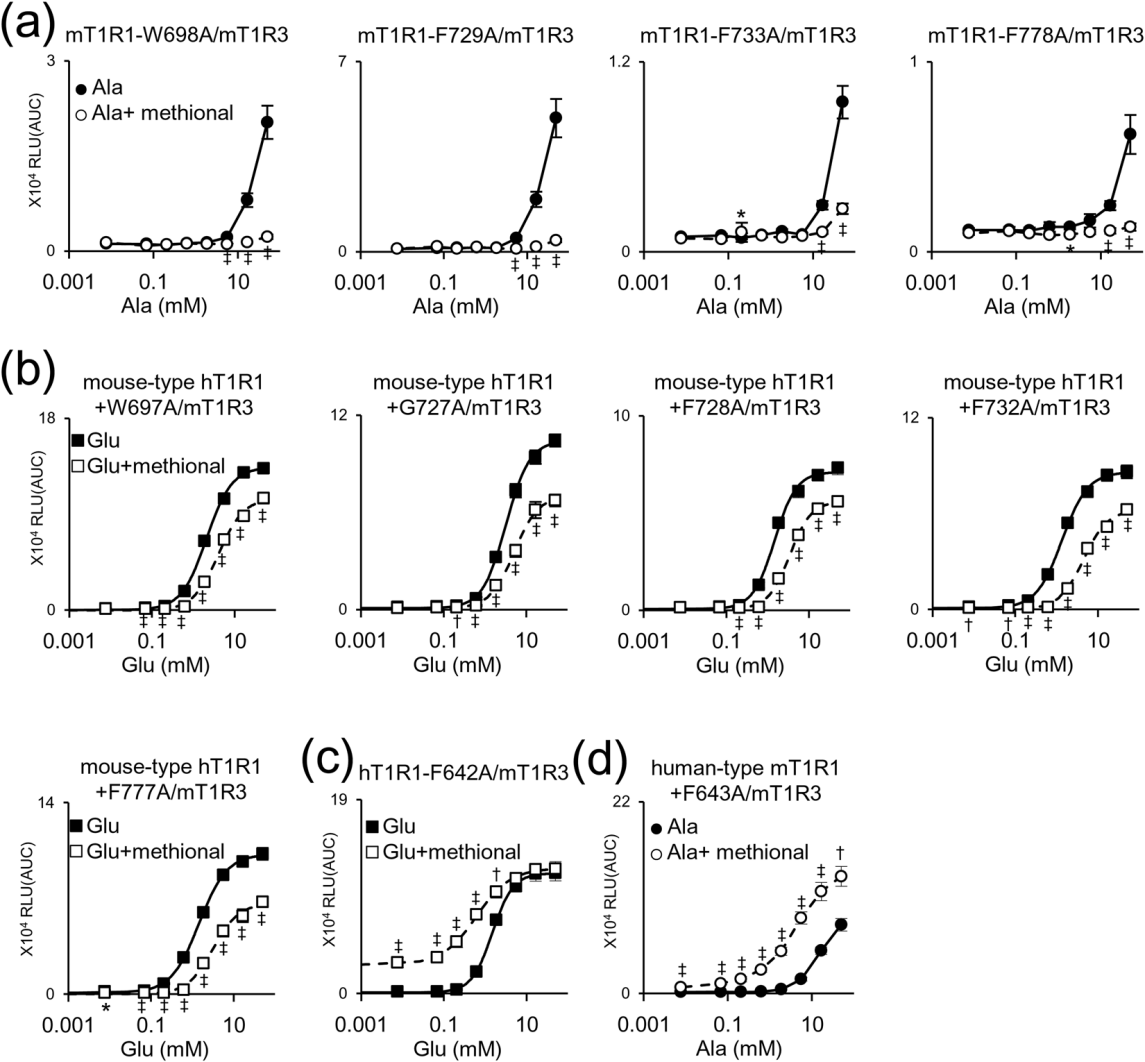
Distinct sets of residues conferred either the PAM or NAM activity of methional. (**a**,**b**) The residues described in Fig. 5a did not affect the activity of methional as a NAM. Dose-dependent responses were obtained to l-Ala for each mouse T1R1 mutant (**a**) and to l-Glu for each mouse-type human T1R1 mutant (**b**) paired with mouse T1R3. (**c**,**d**) The residue described in Fig. 5b did not affect the activity of methional as a PAM. Dose-dependent responses were obtained to l-Glu for the human T1R1 mutant (**c**) and to l-Ala for the human-type mouse T1R1 mutant (**d**) paired with mouse T1R3. Significant differences between l-amino acid responses with and without 120 μM methional were analyzed using Student’s *t* test (*p < 0.05; ^†^*p* < 0.01; ^‡^*p* < 0.001). Values represent the mean ± SE of the RLU (AUC) of 6 recorded wells. The EC_50_ and *E*_max_ values of the receptors described in b and c are shown in Supplementary Tables S1 and S2, respectively.

To examine whether these six residues also affected the activity of methional as a NAM, we introduced an alanine mutation at each of the corresponding residues in both wild-type mouse T1R1 and mouse-type human T1R1. In these mutant receptors paired with mouse T1R3, methional retained its activity as a NAM (Fig. 6a,b, and Supplementary Table S2), except in three receptors (mT1R1-L620A, mT1R1-G728A, and the mouse-type hT1R1 + L619A) that were nonfunctional in our assay and exhibited no detectable responses even to l-amino acids. Thus, to identify the residues that are crucial to NAM activity, we re-scanned all of the target residues by introducing an alanine mutation into the mouse-type hT1R1 in which methional served as a NAM (Fig. 3d-2). Although methional retained PAM activity in the corresponding mutant receptors of both wild-type hT1R1 and human-type mT1R1 (Fig. 6c,d, and Supplementary Table S1), the F642A mutation in mouse-type hT1R1 resulted in a loss of methional activity as a NAM and gain of activity as a weak PAM (Fig. 5b and Supplementary Table S2). Alanine-scanning mutagenesis also revealed that two point mutations (F638A and W773A) in human T1R1 converted methional from a PAM to a NAM (Fig. 5c and Supplementary Table S1).

### Docking of methional to the TMD of T1R1

**Figure 7:**
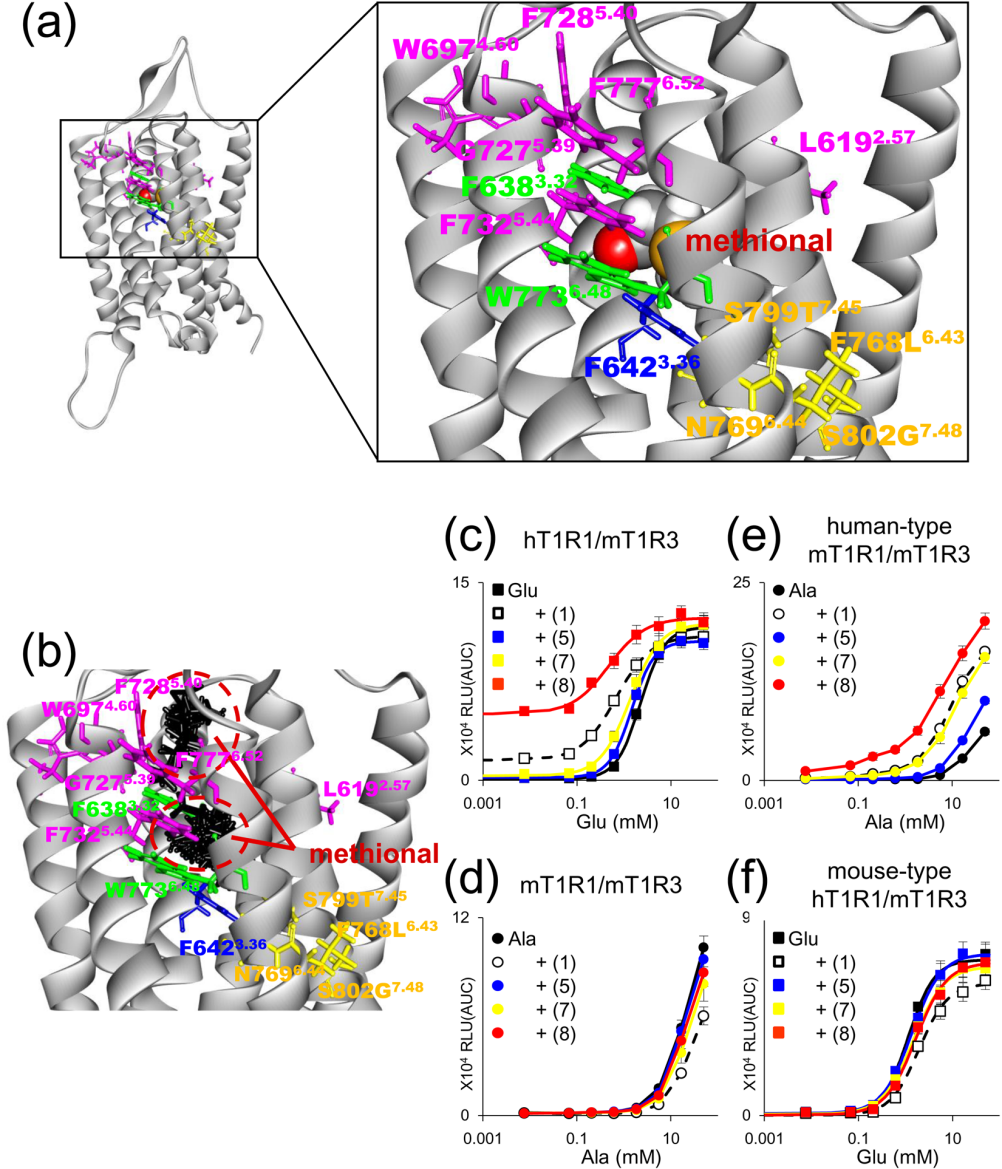
Widespread areas engendering the PAM/NAM activities of methional. (**a**,**b**) Two distinct putative binding sites for methional. A methional molecule docked into the homology model of the transmembrane domain of mouse-type hT1R1. The residues that confer PAM activity are represented in magenta, those that confer NAM activity are represented in blue, and those that contribute to PAM/NAM mode-switching are represented in yellow. Two residues in which an alanine substitution changed the activity of methional from that of a PAM to that of a NAM are represented in green. The residues are also numbered according to the Ballesteros-Weinstein numbering scheme^29^. A representative docking pose with the highest Glide docking score is shown (**a**). Methional is shown as a space-filling model. All of the top 50 poses, ranked by the Glide docking score, are superimposed and displayed, and methional molecules are shown in black (**b**). All of the 40 top poses showed that methional binds to the lower region of the allosteric pocket, while 8 of the top poses ranked from 41 to 50 suggested a binding site in the upper region of the allosteric pocket. The dotted red circles represent the upper and lower putative binding regions of methional. (**c**–**f**) Methional was the strongest NAM for the mouse-type T1R1 receptor, while 3-(methylthio)butanal **(8)** was the strongest PAM for the human-type receptor. Dose-dependent responses to l-Glu (**c**,**f**) or l-Ala (**d**,**e**) were obtained in the presence and absence of methional and each of its analogs (120 μM). Values represent the mean ± SE of the RLU (AUC) of 6 recorded wells. The EC_50_ and *E*_max_ values of the receptors described in c and f are shown in Supplementary Table S3.

Based on the X-ray crystal structure of mGluR1^24^, we created a homology model of the TMD of T1R1. As the template structure was bound to the NAM, we used mouse-type hT1R1, in which methional serves as a NAM, to construct the structural model (Fig. 3d-2). This model indicated that all four residues that primarily contribute to switching the PAM/NAM activities of methional (L768, N769, T799, and G802 in the mouse-type hT1R1) (Fig. 3d) were clustered at the middle of the TMD (Fig. 7a). The NAM activity-conferring residue, F642 (Fig. 5b), was located near the PAM/NAM mode-switching site. By contrast, all six PAM activity-conferring residues (L619, W697, G727, F728, F732, and F777) (Fig. 5a) were positioned at the upper site of the TMD. Two residues, F638 and W773, for which alanine substitutions in hT1R1/mT1R3 converted methional from a PAM to a NAM (Fig. 5c), were located at the border of the PAM and NAM activity-conferring sites.

We docked a methional molecule into our model using Glide software. All of the top 40 poses based on the Glide docking score showed that methional bound at the lower region of the allosteric pocket (Fig. 7a,b). These models showed that methional was located near the NAM activity-conferring residue that we identified, while most of the PAM activity-conferring residues were far from the docked position of methional. By contrast, among the top 50 poses, 8 lower-ranking poses indicated that methional could bind at a distinct site located in the upper region of the allosteric site (Fig. 7b).

To further evaluate binding site candidates, we compared the activities of structural analogs of methional when they acted as PAMs or NAMs. Although 3-(methylthio)butanal **(8)** was the strongest PAM for hT1R1/mT1R3 (Fig. 7c and Supplementary Table S3) and hT1R1/hT1R3 (Fig. 1j), methional **(1)** exhibited the strongest NAM activity for mT1R1/mT1R3 (Fig. 7d). Similarly, 3-(methylthio)butanal **(8)** exhibited the strongest PAM activity for human-type mT1R1/mT1R3 (Fig. 7e), while methional **(1)** was the strongest NAM for mouse-type hT1R1/mT1R3 (Fig. 7f and Supplementary Table S3). The different affinities of the structural analogs between the PAM and NAM modes suggested that the binding positions of these modulators differ when they serve as PAMs or NAMs.

## Discussion

In this study, we demonstrate that methional and its structural analogs serve as allosteric/ago-allosteric modulators for T1R1/T1R3 through the contribution of widespread regions in the TMD of T1R1. There have been no reports on the molecular mechanisms underlying the allosteric modulation in the TMD of T1R1. By focusing on the unique species-dependent difference in the methional activity, we could reveal the mechanism of the positive and negative modulation in T1R1.

As methional has a pleasant meaty and soup-like flavor^25^, its aroma has been reported to evoke an umami sensation via a cross-modal interaction between taste and smell^13,14^. However, our results demonstrate that methional directly affected umami taste via peripheral gustatory receptors in addition to its effects via the olfactory system. Recently, Suess *et al*. also reported that an odorant reduced the bitterness of caffeine by interacting with bitter taste receptors (TAS2Rs)^26^.

Analysis of structural analogs suggested that the optimum structure to activate hT1R1/hT1R3 is an aldehyde with a methylthio group at C-3 (Fig. 1). Although 5′-ribonucleotides do not activate hT1R1/hT1R3 on their own^5^, methional and 3-(methylthio)butanal **(8)** function as both PAMs and weak agonists for hT1R1/hT1R3 (Fig. 1a,k). Methional and 3-(methylthio)butanal **(8)** can enhance l-Glu responses even when applied together with IMP (Fig. 2), implying that their binding site is distinct from that of IMP, which is located within the extracellular Venus flytrap domain (VFTD) of T1R1^7^.

To our knowledge, methional is the first-known compound that acts as both a PAM for the human taste receptor and NAM for the rodent receptor (Fig. 3). Analysis using chimeric receptors and point mutants demonstrated that the TMD of T1R1 is the key domain for switching the PAM/NAM activities of methional and identified four residues (h/m; F768/L769, N769/H770, S799/T800, and S802/G803) that were collectively sufficient to switch PAM/NAM activities (Fig. 3). A similarly drastic mode change in allosteric modulator activity has been reported in site-directed mutagenesis studies of a related GPCR, metabotropic glutamate receptor 5 (mGluR5)^27,28^. N769 and S799 in human T1R1 correspond to the residues in rat mGluR5 (Fig. 4) in which the introduction of an alanine mutation converted its PAMs to NAMs^27^. Moreover, S799 and S802 in human T1R1 correspond to the residues in T1R3 that reportedly contribute to the activities of sweeteners (NHDC and cyclamate) and/or a sweet taste inhibitor (lactisole) (Fig. 4)^21–23^. Winnig *et al*. proposed that C801 in T1R3, which corresponds to S799 in human T1R1 (Fig. 4), plays a general role in the activation process of the sweet taste receptor rather than directly interacting with ligands because it is far from the binding site of lactisole^23^. Thus, we hypothesize that the four residues identified here mainly affect a global conformation of T1R1/T1R3 that induces the PAM/NAM mode switch rather than directly interacting with methional.

To identify the residues that confer the PAM and NAM activities of methional, we performed alanine-scanning mutagenesis of the corresponding residues that are crucial to the activities of NHDC, cyclamate, and lactisole in human T1R3. As a result, we identified six residues (L619, W697, G727, F728, F732, and F777 in hT1R1) that confer the PAM activity of methional (Fig. 5a). These results validate our hypothesis that different sets of residues contribute to either engendering the PAM/NAM mode switch or conferring the PAM/NAM activity of methional. Moreover, these residues did not affect the activity of methional as a NAM (Fig. 6a,b, and Supplementary Table S2), implying that additional residues contribute to imparting the NAM activity of methional. In fact, alanine-scanning mutagenesis using the mouse-type hT1R1 identified that F642 is crucial to the NAM activity (Fig. 5b and Supplementary Table S2), but did not affect the PAM activity of methional (Fig. 6c,d, and Supplementary Table S1). These results suggest that both the PAM and NAM activities of methional are conferred by residues that are distinct from those engendering the PAM/NAM mode switch.

A homology model of the TMD of mouse-type hT1R1 indicated that all four residues that primarily contribute to switching the PAM/NAM activities of methional (L768, N769, T799, and G802 in the mouse-type hT1R1) are clustered at the middle of the TMD (Fig. 7a). This position is at the bottom edge of the putative allosteric binding site, which was predicted by alignment with other GPCRs^21–24^. The NAM activity-conferring residue F642 (Fig. 5b) is located near the PAM/NAM mode-switching site. By contrast, all six PAM activity-conferring residues (L619, W697, G727, F728, F732, and F777) (Fig. 5a) are positioned at the upper site of the TMD. L619 is located away from the other five residues, possibly explaining its different contribution to the efficacy of l-Glu (Fig. 5a and Supplementary Table S1). Two residues, F638 and W773, for which alanine substitutions in hT1R1/mT1R3 converted methional from a PAM to a NAM (Fig. 5c), are located at the border of the PAM and NAM activity-conferring sites. W773 (W6.48 numbered according to the system of Ballesteros and Weinstein^29^) is widely conserved among GPCRs and is thought to function as a transmission switch for receptor activation^30^. Moreover, the Phe residue that faces W6.48 in TM-3 has been reported to act as a key switch residue in the activation process of a class A GPCR, the cannabinoid CB1 receptor^31^. Our mutagenesis experiment also suggested that W6.48 and the Phe residue in the TM-3 function as a microswitch to activate T1R1/T1R3, which is induced by a modulator that interacts with the TMD.

The docking models suggested two putative binding sites for methional at the upper and lower regions of the allosteric pocket (Fig. 7b). Because a crystal structure of the TMD in complex with a PAM is not currently available for class C GPCRs, we could not fully define the docked pose of methional as a PAM. However, taken together with the results that PAM activity-conferring residues were clustered in the upper region of the allosteric site, these data suggest that this upper area is a potential binding site for methional when it serves as a PAM. The binding pocket consists of many hydrophobic residues that face inside, and most of the crucial residues to the methional activity were also hydrophobic amino acids (Fig. 7). Methional is amphiphilic compound and its LogP value is −0.16. Therefore, there is a strong possibility that methional binds to both the upper and lower regions of the TMD of T1R1. Additionally, 3-(methylthio)butanal **(8)** is hypothesized to bind at the upper binding site rather than the lower site because it was the bulkiest analog used in this study. This is likely one of the reasons why 3-(methylthio)butanal **(8)** acts as a strong PAM for human T1R1/T1R3 but a weak NAM for mouse T1R1/T1R3 (Fig. 7c–f). As an alanine mutation at each of PAM activity-conferring residues also resulted in decreased activity of methional as an agonist (Figs 3b-2 and 5a), methional could also bind at the same upper binding site as when it served as a PAM.

**Figure 8:**
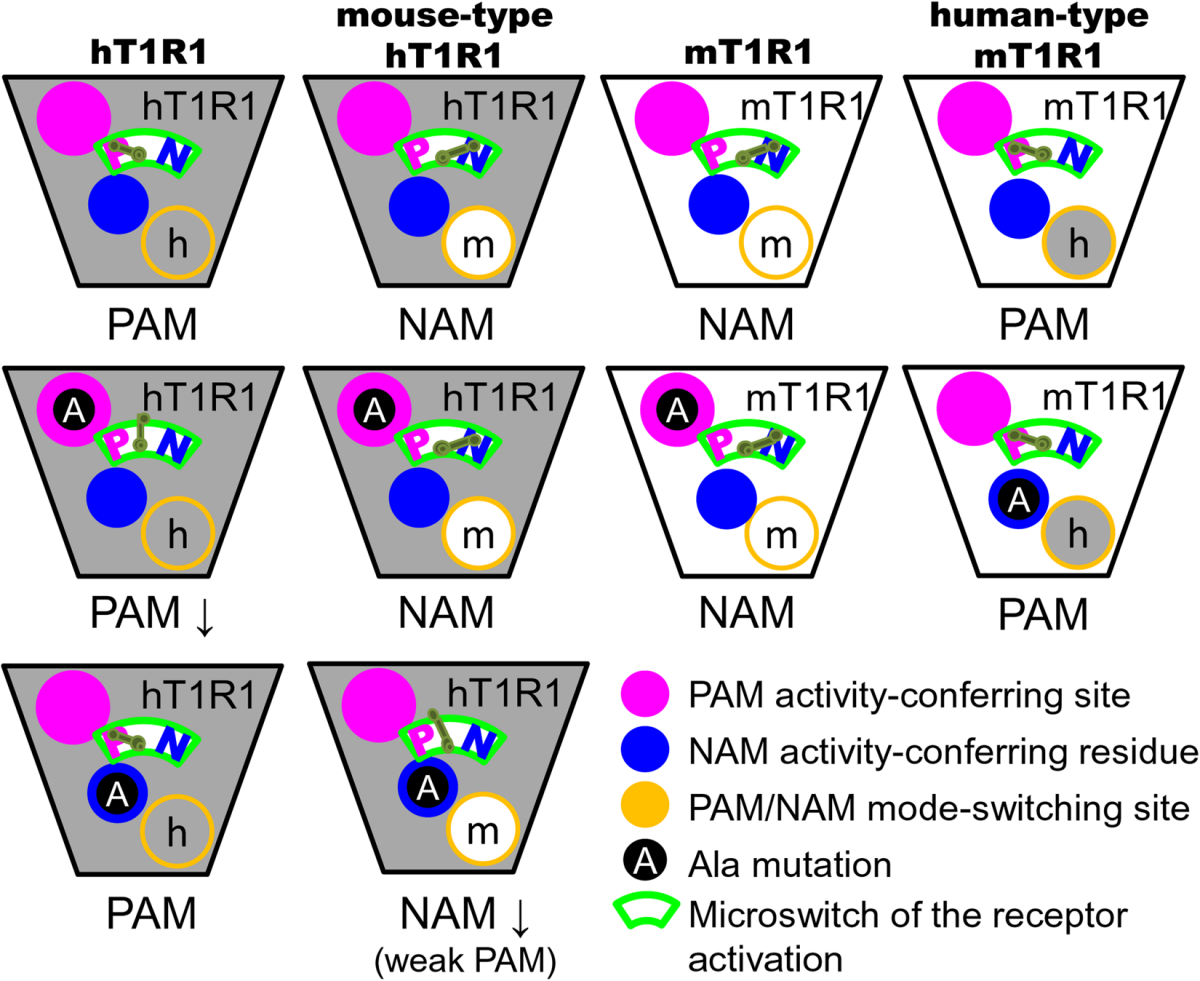
Cartoon representing a summary of the receptor expression experiments described in this paper. The PAM/NAM modes of methional primarily depended upon whether the residues at the bottom of the binding site were from the human or mouse amino acid sequences. PAM and NAM activities were conferred by distinct sites that were located at the upper or the lower regions, respectively, of the allosteric pocket across the microswitch responsible for receptor activation.

Owing to the small molecular size of methional, we could not assume that the residues that we identified either coordinate a direct interaction with methional or have a global effect on protein conformations for engendering active/negative conformations. However, our data may suggest an attractive mechanism by which a small-molecule compound, methional, is endowed with PAM/NAM activities by widespread regions in the allosteric site that have distinct effects on the activity of methional. Two distinct putative binding sites for methional exist across the microswitch for receptor activation, and the residues at the bottom of the allosteric site might contribute to switching the binding positions of methional (Fig. 8). GPCRs are involved in most pathophysiological processes and are the targets of many therapeutic agents^32^. These findings could contribute to understanding the molecular basis of the allosteric modulations in T1Rs and other GPCRs.

Methional has been used as an essential flavor additive for various processed foods, such as gravies, meat products, baked goods, and condiments, at a wide range of concentrations (0.6–120 μM)^25^. Our results suggest that the widespread use of methional might be due not only to its pleasant aroma but also its preferable effect on the sensation of umami taste.

In addition to the use of methional as a food additive, various foods naturally contain methional via degradation of methionine^25^. Foods that are used globally as seasonings, such as tomatoes^8,9^, cheese^10,11^, and soy sauce^12^, commonly contain methional as one of their main flavor components. Among the structural analogs that we used, 2-methylthioacetaldehyde **(5)** and 3-(methylthio)butanal **(8)** are also reported in natural products, such as tomatoes and krill^25^. Our receptor assay showed that methional activated T1R1/T1R3 in conjunction with other umami tastants, such as amino acids and 5′-ribonucleotides (Fig. 2). In fact, these foods also commonly contain high concentrations of l-Glu^33^, which is the most common substance conferring an umami taste for humans. Moreover, the concentration of methional is higher in heated than fresh tomatoes^34^. Thus, in cooperation with amino acids and 5′-ribonucleotides, methional could contribute to enhancing the umami taste of our meals through cooking.

## Methods

### Materials

Samples were obtained from commercial sources as follows: l-aspartic acid sodium salt, l-glutamic acid monosodium salt, l-serine, l-lysine monohydrochloride, l-histidine monohydrochloride monohydrate, l-proline, and l-phenylalanine were purchased from Nacalai Tesque; l-glutamine, l-threonine, glycine, l-alanine, l-valine, l-isoleucine, l-leucine, l-arginine, and l-asparagine monohydrate were obtained from Kanto Chemical; l-methionine, methionol, pentanal, 3-(methylthio)propionic acid, and 3-(methylthio)butanal were obtained from Tokyo Chemical Industry; methional was from Sigma Aldrich; 2-methylthioacetaldehyde was from Penta Manufacturing Company; 3-(ethylthio)propanal was from Enamine Ltd.; 4-(methylthio)butanal was from FCH Group; and coelenterazine was purchased from Promega.

### Constructs for Human-Rodent Chimeric Taste Receptors and their Point Mutants

hT1R1 (NCBI RefSeq number NM_138697.3), hT1R3 (NM_152228.1), mT1R1 (NM_031867.2), mT1R3 (NM_031872.2), human-mouse T1R1 chimeras, and point mutants of hT1R1 and mT1R1 were constructed by polymerase chain reaction (PCR) using overlapping primers as previously described^15^ and were subcloned into the pEAK10 expression vector (Edge Biosystems) at the *Asc* I-*Not* I site. The Kozak consensus sequence was introduced upstream of the start codon for efficient translation. For alanine scanning mutagenesis, targeted alanine residues were mutated to other bulky or charged amino acids (hT1R1-A639V, hT1R1-A639H, hT1R1-A731V, hT1R1-A731E, hT1R1-A780V, hT1R1-A780Y, and hT1R1-A795L).

### Luminescence-based Assay for T1R1/T1R3

T1R responses were measured in heterologous cells using a luminescence-based assay, as previously described^15^. HEK293T cells were transiently co-transfected with expression vectors for T1R1, T1R3, hG16gi3, and mt-apoaequorin and, after 48 h of transfection, exposed to test stimuli and assayed for luminescence. Methional and its analogs were dissolved in DMSO to 240 mM and then diluted to their desired concentrations in assay buffer. Control solutions were prepared by matching the DMSO concentration to that of the test solutions. The response from each well was calculated based on the area under the curve (AUC) and is expressed as relative light units (RLU). For receptors in which the responses to l-amino acids were saturated at the highest concentration tested (50 mM), plots of the amplitudes versus concentrations were fitted to the Hill equation, and the EC_50_ values and *E*_max_ values were evaluated (Supplementary Table S1, S2, and S3). Statistical analysis was performed using Student’s *t*-test and one-way ANOVA followed by Tukey’s test using the software Ky Plot version 3.0.

### Homology Model Development

A homology model of the mouse-type human T1R1 was created using Prime (Schrödinger, LLC) and based on an inactive form of the metabotropic glutamate receptor 1 (mGluR1) (PDB ID: 4OR2), which is one of the most closely related receptors to the T1Rs^24^.

### Glide Docking of Methional

The SiteMap program (Schrödinger, LLC) was used to initially identify two potential ligand binding sites for the mouse-type human T1R1; one was near the three mutated residues at the bottom of the binding site, and the other was in the upper region of the allosteric pocket. Glide docking (Schrödinger, LLC) was then performed to allow methional to explore both of these potential binding sites. Standard precision (SP) mode and the OPLS3 force field were used during docking.

### Data availability

All data generated or analyzed during this study are available from the corresponding author on reasonable request.

## Electronic supplementary material

Supplementary Information

Supplementary PDB files
