# Supplementary material for: Positive/Negative Allosteric Modulation Switching in an Umami Taste Receptor (T1R1/T1R3) by a Natural Flavor Compound, Methional
Source: Sci Rep. 2018 Aug 7;8:11796. doi: 10.1038/s41598-018-30315-x (PMC6081381; doi:10.1038/s41598-018-30315-x)
Supplement: Supplementary file 1 — Supplementary Information [file 41598_2018_30315_MOESM1_ESM.doc]

**Supplementary Information**

Positive/Negative Allosteric Modulation Switching in an Umami Taste Receptor (T1R1/T1R3) by a Natural Flavor Compound, Methional

Yasuka Toda^1, 2^, Tomoya Nakagita^1, 3^, Takatsugu Hirokawa^4^, Yuki Yamashita^5^, Ayako Nakajima^5^, Masataka Narukawa^1^, Yoshiro Ishimaru^2^, Riichiro Uchida^5^, and Takumi Misaka*^1^

^1^ Department of Applied Biological Chemistry, Graduate School of Agricultural and Life Sciences, The University of Tokyo, 1-1-1 Yayoi, Bunkyo-ku, Tokyo 113-8657, Japan

^2^ Department of Agricultural Chemistry, Faculty of Agriculture, Meiji University, 1-1-1 Higashimita, Tama-ku, Kawasaki, Kanagawa 214-8571, Japan

^3^ Department of Cell Biology, Graduate School of Medicine, Kyoto University, Yoshida-Konoe-cho, Sakyo-ku, Kyoto 606-8501, Japan

^4^ Molecular Profiling Research Center for Drug Discovery (molprof), National Institute of Advanced Industrial Science and Technology (AIST), Tokyo Waterfront Bio-IT Research Building 2-4-7 Aomi, Koto-ku, Tokyo 135-0064, Japan.

^5^ Research and Development Division, Kikkoman Corporation, 399 Noda, Noda, Chiba 278-0037, Japan

**Supplementary Table S1.** Effect of alanine substitutions on the l-Glu responses in hT1R1/mT1R3. Values represent the mean ± SE of the RLU(AUC) of 6 recorded wells.

|  | EC_50_ (mM) | | | Relative EC_50_ | *E*_max_ (X10^4^ RLU(AUC)) | | | Relative *E*_max_ |
| --- | --- | --- | --- | --- | --- | --- | --- | --- |
|  | Glu | + 120 µM methional | | % of Glu EC_50_ | Glu | + 120 µM methional | | % of Glu *E*_max_ |
| hT1R1-WT/mT1R3 | 1.8 ± 0.2 | 0.6 ± 0.1 | *** | 36 | 14.2 ± 0.7 | 14.1 ± 0.6 |  | 99 |
| L619A | 13.7 ± 1.9 | 7.5 ± 0.8 | * | 55 | 4.2 ± 0.5 | 2.8 ± 0.3 | * | 66 |
| W697A | 3.3 ± 0.2 | 2.7 ± 0.1 |  | 82 | 8.9 ± 0.7 | 8.8 ± 0.5 |  | 98 |
| G727A | 6.7 ± 1.1 | 4.0 ± 0.5 | *p=0.05* | 61 | 14.5 ± 0.7 | 15.1 ± 0.8 |  | 104 |
| F728A | 2.3 ± 0.1 | 2.6 ± 0.1 |  | 113 | 9.2 ± 0.4 | 8.8 ± 0.4 |  | 96 |
| F732A | 2.3 ± 0.3 | 1.8 ± 0.1 |  | 78 | 8.9 ± 0.3 | 8.5 ± 0.6 |  | 95 |
| F777A | 2.6 ± 0.1 | 1.7 ± 0.1 | *** | 65 | 16.3 ± 0.5 | 15.9 ± 0.5 |  | 98 |
| F638A | 2.1 ± 0.2 | 4.0 ± 0.3 | *** | 188 | 11.7 ± 1.1 | 8.5 ± 0.5 | * | 72 |
| W773A | 4.2 ± 0.3 | 7.2 ± 0.6 | ** | 173 | 15.2 ± 0.5 | 11.7 ± 0.6 | *** | 77 |
| F642A | 1.5 ± 0.1 | 0.6 ± 0.1 | *** | 42 | 11.9 ± 0.8 | 12.3 ± 0.6 |  | 103 |

*, *p* < 0.05; **, *p* < 0.01; ***, *p* < 0.001 vs. l-Glu, Student′s *t* test.

**Supplementary Table S2.** Effect of alanine substitutions on the l-Glu responses in mouse-type hT1R1/mT1R3. Values represent the mean ± SE of the RLU(AUC) of 6 recorded wells.

|  | EC_50_ (mM) | | | Relative EC_50_ | *E*_max_ (X10^4^ RLU(AUC)) | | | Relative *E*_max_ |
| --- | --- | --- | --- | --- | --- | --- | --- | --- |
|  | Glu | + 120 µM methional | | % of Glu EC_50_ | Glu | + 120 µM methional | | % of Glu *E*_max_ |
| mouse-type hT1R1-WT/mT1R3 | 0.8 ± 0.0 | 1.2 ± 0.1 | ** | 159 | 10.5 ± 0.5 | 9.1 ± 0.4 |  | 87 |
| + L619A | n. d. | | | n. d. | n. d. | | | n. d. |
| + W697A | 2.1 ± 0.0 | 4.1 ± 0.4 | *** | 193 | 13.5 ± 0.3 | 10.6 ± 0.6 | ** | 78 |
| + G727A | 3.4 ± 0.2 | 6.3 ± 0.9 | * | 185 | 10.6 ± 0.4 | 7.4 ± 0.4 | *** | 70 |
| + F728A | 1.5 ± 0.1 | 3.6 ± 0.3 | *** | 241 | 7.1 ± 0.3 | 5.6 ± 0.2 | ** | 79 |
| + F732A | 1.4 ± 0.1 | 4.8 ± 0.5 | *** | 343 | 8.7 ± 0.4 | 6.4 ± 0.4 | ** | 73 |
| + F777A | 1.5 ± 0.1 | 3.7 ± 0.6 | ** | 250 | 10.3 ± 0.4 | 6.8 ± 0.3 | *** | 66 |
| + F642A | 2.7 ± 0.2 | 2.1 ± 0.1 | * | 78 | 19.2 ± 1.2 | 16.8 ± 1.0 |  | 88 |

n.d., not determined.

*, *p* < 0.05; **, *p* < 0.01; ***, *p* < 0.001 vs. l-Glu, Student′s *t* test.

**Supplementary Table S3.** Activity of methional and its structural analogs in hT1R1/mT1R3 and mouse-type hT1R1/mT1R3. Values represent the mean ± SE of the RLU(AUC) of 6 recorded wells.

| hT1R1-WT/mT1R3 | EC_50_ (mM) | | *E*_max_ (X10^4^ RLU(AUC)) | |
| --- | --- | --- | --- | --- |
| Glu | 2.3 ± 0.1 |  | 11.7 ± 0.7 |  |
| + **(1)** | 0.7 ± 0.1 | *** | 10.9 ± 0.5 |  |
| + **(5)** | 1.5 ± 0.1 | *** | 10.5 ± 0.5 |  |
| + **(7)** | 1.4 ± 0.1 | *** | 11.9 ± 0.7 |  |
| + **(8)** | 0.5 ± 0.1 | *** | 12.4 ± 0.6 |  |
| mouse-type hT1R1/mT1R3 | EC_50_ (mM) | | *E*_max_ (X10^4^ RLU(AUC)) | |
| Glu | 1.1 ± 0.1 |  | 7.0 ± 0.5 |  |
| + **(1)** | 2.0 ± 0.1 | *** | 6.0 ± 0.4 |  |
| + **(5)** | 1.4 ± 0.2 |  | 7.4 ± 0.7 |  |
| + **(7)** | 1.5 ± 0.1 |  | 6.8 ± 0.7 |  |
| + **(8)** | 1.7 ± 0.1 | * | 7.0 ± 0.5 |  |

*, *p* < 0.05, **, *p* < 0.01, ***, *p* < 0.001 vs. l-Glu, Student′s *t* test.

**Supplementary Figure Legends**

**Supplementary Fig. S1.** A methional molecule docked into the homology model of the transmembrane domain of mouse-type hT1R1. The docking pose with the highest Glide docking score when methional binds at the upper site is shown.

**Supplementary Fig. S2.** A methional molecule docked into the homology model of the transmembrane domain of mouse-type hT1R1. The docking pose with the highest Glide docking score when methional binds at the lower site is shown.
